# Outcomes of liver transplantation for hepatocelluler carcinoma from living donor versus deceased donor within University of Southern California San Francisco criteria: a report from Turkey

**DOI:** 10.3389/fonc.2024.1419740

**Published:** 2024-08-22

**Authors:** Imam Bakır Batı, Umut Tüysüz

**Affiliations:** ^1^ Department of Liver Transplant Surgery, Faculty of Medicine, Acıbadem University, Istanbul, Türkiye; ^2^ Department of Liver Transplant Surgery, Şişli Etfal Hamidiye Training and Research Hospital, Istanbul, Türkiye

**Keywords:** hepatocellular cancer, living donor liver transplantation, deceased donor liver transplantation, recurrence, survival

## Abstract

**Background:**

Hepatocellular cancer (HCC) is the most common primary liver cancer with increasing incidence. Liver transplantation (LT) has been accepted as main curative liver cancer treatment. The effectiveness of LDLT as opposed to Deceased Donor Liver Transplant (DDLT) for patients with HCC is still controversial. There is limited data comparing the long-term outcomes of patients undergoing LDLT or DDLT for HCCs that do not meet the Milan criteria.

**Methods:**

We aimed to compare the perioperative and survival outcomes of LDLT with DDLT in HCC patients.Patients underwent LT between January 2012 and December 2020 were retrospectively analyzed. There were 137 patients who met the UCSF criteria. Of these, 75 patients received LDLT and 62 patients DDLT.The primary end points in the present study were oncologic outcomes such as the recurrence rate, disease-free survival (DFS) and overall survival (OS) of LDLT and DDLT in patients with HCC.

**Results:**

PET-CT SUVmax value, the amount of erythrocyte solution (ES) as blood transfusion of red cells given and the tumor recurrence rate were significantly higher among the deceased patients recurrence, ES, PET-CT SUVmax value and tumor differentiation had significant effects on survival. In the multivariate reduced model, cox regression analysis showed significant effects of recurrence, ES, locoregional treatment response and PET-CT on survival.Albeit not significant, the one-year recurrence rate in the LDLT was similar to that in the DDLT, three- and five-year recurrence rates were higher in DDLT compared to LDLT

**Conclusion:**

There is less chance of cold ischemia time and better-quality grafts with minimal fatty changes, lower recurrence rates and similar survival rates can be achieved in LDLT compared to DDLT

## Introduction

Hepatocellular cancer(HCC) is the 7^th^ most common cancer among malignancies and the 3^rd^ most common cancer among cancer-related deaths in the world (1). İt is the most common primary liver cancer with increasing incidence and estimated death rate of 55% until 2040 (2). Although local ablation and resection are considered as potential curative treatments, the limited functional reserve of the liver restricts their applicability. In addition, the chance of recurrence of the disease is high in reserve liver tissue (3). Since the development of the Milan criteria in 1996, liver transplantation (LT) has been accepted as main curative liver cancer treatment (4), because it potentially treats both the tumor and the underlying liver disease. However, the majority of Living Donor Liver Transplant (LDLT) centers have adopted the latest expanded Milan/University of Southern California San Francisco (UCSF) criteria. The effectiveness of LDLT as opposed to Deceased Donor Liver Transplant (DDLT) for patients with HCC is still controversial. One study showed inferior oncological outcomes after LDLT compared to deceased donor transplantation. Patients undergoing LDLT had higher HCC recurrence rates within 3 years (5). Another analysis later supported this findings. Authors observed a higher recurrence after LDLT, which could have been due to different tumor characteristics and HCC management before transplantation (6). A systematic literature review and meta-analysis reported an inferior disease- free survival (DFS) after LDLT when compared to deceased donation (7). We also aim to underline the need for and improved study design and reporting to understand if the observed DFS difference might be attributed to a study bias or are the result of other contributors in context of LDLT

Compared to conventional DDLT, LDLT significantly shortens the waiting time for appropriate grafts and is widely accepted as an alternative treatment for end-stage liver disease. Since 1989, when the first successful living donor liver transplantation was performed, LDLT has been a reliable alternative to DDLT by reducing rates of drop from the waiting list. Because live donor grafts are not limited by the constraints of the national organ distribution system, LDLT indications for patients with HCC are often case dependent and related to institutional considerations, the recipient’s overall survival benefit and operative risks, and broad criteria are easily applied without breaking the equation (8). However, the debate on the spread of LDLT for HCC patients continues. If patient selection is carried out through strictly adhering to Milan criteria, the five-year survival rate is between 70 and 90%. A number of studies proved that the Milan criteria are very restrictive and that more acceptable results can be achieved with more liberal selection criteria. Expanded criteria were first proposed by the UCSF group (9). However, there is limited data comparing the long-term outcomes of patients undergoing LDLT or DDLT for HCCs that do not meet the Milan criteria. In the present study, at the same time, we aimed to compare the perioperative and survival outcomes of LDLT with DDLT in HCC patients.

## Methods

This was a retrospective study from Acıbadem hospital Bursa, and Acıbadem Hospital, University of Acıbadem, Turkey. All data were taken prospectively from the stored database. Medical records of 1,016 patients who underwent LT between January 2012 and December 2020 were retrospectively analyzed. There were 178 patients with histologically confirmed HCC. Twenty patients had multiple tumors. The main tumor size was out of the criteria in 11 patients. The explant pathology was reported as combined type (HCC and intrahepatic cholangiocarcinoma) in 10 patients. When these were excluded, there were 137 patients who met the UCSF criteria. The study intends to investigate all-inclusive HCC within UCSF (9). DDLT was performed with grafts from brain-dead donors using piggyback techniques for whole LT. The type of graft (ie, whole liver or split liver) was not defined in advance. All of LDLT procedures were performed with right liver grafts from living donors. Operative mortality was defined as death either during the perioperative period of hospitalization for LT or up to 90 days after LT. Early operative morbidity was defined as within 30 days (ie.biliary leak,arter-vein complications). They were excluded from study. All of the recipients received standard immunosuppression protocol (steroids + mycophenolate mofetil + tacrolimus) after LT. If needed, the protocol was added with mammalian target of rapamycin inhibitors. (mTOR). Patients did not undergo pre and postoperative chemo- or radiotherapy. These, 75 patients received LDLT and 62 patients DDLT. All patients with an expected waiting list time longer 3 months were treated with transarterial chemoembolization (TACE), ablation as pretransplant bridging therapy. Downstaging therapies before transplantation include TACE and ablation. We used downstaging to bring the patients in UCSF group. When Any of the patient which was down staged fall within UCSF, they were excluded from the study. The decision to treat and the type of treatment were discussed in multidisciplinary meeting. All patients were prioritized according to the Model for End Stage Liver Diseases score (MELD) (10). Tumor size and number were investigated using pathologic outcomes. Explant livers were examined for tumor size, number, differentiation, and microvascular invasion by specialist pathologists. Tumor evaluation was based on contrast imaging such as computed tomography (CT), magnetic resonance imaging (MRI) and Fluorodeoxyglucose-positron emission tomography (FDG-PET). Patient follow-up protocols after LT included AFP and computed tomography and/or magnetic resonance imaging were performed every 3 months in the first year after liver transplantation and then every 6 months. MRI was performed if a follow-up CT examination suggested recurrence. If extrahepatic recurrence was suspected based on clinical symptoms or unexplained elevation of tumor marker levels, patients underwent chest CT and positron emission tomography scanning with 18F-fluorodeoxyglucose. Recurrence-free survival (RFS) was measured from the date of LT until HCC recurrence (intra- or extrahepatic) or until the final documented date of no evidence of tumor recurrence on imaging studies. Overall survival (OS) was defined as the interval between LT and death or the date of the final follow-up visit. Blood transfusion referred to the transfusion of packed red blood cells during excessive intraoperative bleeding or postoperative bleeding complications. Transfusions of fresh-frozen plasma (FFP) were also included.

The primary end points in the present study were oncologic outcomes such as the recurrence rate, disease-free survival (DFS) and overall survival (OS) of LDLT and DDLT in patients with HCC.

### Statistical analyses

Mean, standard deviation, median, minimum, maximum value, frequency and percentage were used for descriptive statistics. The distribution of variables was checked with Kolmogorov-Smirnov test.Mann-Whitney U test was used for the comparison of quantitative data. Chi-Square test was used to compare the qualitative data.Cox regression and Kaplan-Meier test were used in the survival analysis. Uni- and multivariable analyses to identify factors associated with RFS and OS were performed by using Cox proportional hazard regression models. HCC recurrence and OS were compared between the two groups in a propensity score-matched cohort using a Log rank test Cumulative recurrence rate and OS rate were calculated by the Kaplan–Meier method. Cox proportional hazard model was used to identify risk factors associated with recurrence. SPSS 27.0 was used for statistical analyses.

## Results

Comparing the surviving and deceased patients, the average age was higher among the patients who died. However, it is known that HCC recurrence is highest within 2-3 years after transplant. Recurrence time also emerges as a prognostic factor. Early HCC recurrence is associated with worse prognosis (11). Considering patients with HCC recurrence, HCC recurrence was observed in 7 of the patients who underwent LDLT, all of which were early recurrence (within 2 years). However, early recurrence has been observed in 8 of 13 patients who underwent DDLT. LDLT were treated 18 patients,DDLT were treated 25 patients as locoregional treatment. Recurrences were liver in 4 patients, liver and bone in 2 patients, lung and liver 1 patient in LDLT group. In DDLT group were liver in 2 patients, liver and bone in 7 patients, liver and lung in 4 patients. Table shows patients demographics, tumor characteristics and operative informations (**Table 1**).

**Table 1:** Clinico-demographic findings.

		Min-Max	Median	Mean±sd/n-%		
Age		37.0 – 77.0	65.0	63.0 ± 7.9		
Gender	Female			25		18.2%
	Male			112		81.8%
BMI		20.0 – 39.0	26.0	26.7 ± 3.7		
Liver Disease						
Budd-Chiari				3		2.2%
Ethanol				4		2.9%
Hepatitis B Virus				77		56.2%
Hepatitis C Virus				23		16.8%
Hemochromatosis				1		0.7%
Criptojenik				21		15.3%
Nash				4		2.9%
Autoımmune				2		1.5%
Wilson				2		1.5%
MELD		0.0 – 32.0	8.0	10.0 ± 6.6		
AFP (ng/ml)		0.3 – 1148.0	8.5	48.5 ± 126.6		
Cold Ischemia (min)		15.0 – 500.0	31.0	148.2 ± 147.1		
TM Diameter (mm)		0.8 – 63.0	26.0	27.3 ± 12.7		
Pet-CT (suv-max)		0.0 – 11.0	2.7	2.6 ± 2.0		
FFP (unit)	1			5		3.6%
	2			97		70.8%
	3			35		25.5%
ES (unit)	1			1		0.7%
	2			36		26.3%
	3			51		37.2%
	4			38		27.7%
	5			11		8.0%
Locoregional Treatment	no			94		68.6%
	yes			43		31.4%
MVI	no			85		62.0%
	yes			52		38.0%
Child-pugh score	A			83		60.6%
	B			49		35.8%
	C			5		3.6%
Tumor Number (lesion)	1			91		66.4%
	2			23		16.8%
	3			22		16.1%
	4			1		0.7%
Tumor Differansiasion	Well			45		32.8%
	Moderately			70		51.1%
	Poorly			22		16.1%
LDLT				75		54.7%
DDLT				62		45.3%
Follow Up time (month)		6.0 – 111.0	48.0	49.9 ± 25.1		

BMI, Body mass ındex; AFP, Alpha Feto Protein FFP, Fresh Frozen Plasma; ES, Eritrosit solution; MVI, Micro vascüler invasion; LDLT, Living donor liver transplantation, DDLT, Deceased donor liver transplantation.

While gender distribution, BMI, MELD score, AFP, cold ischemia time, Child score, degree of tumor differentiation, microvascular invasion, locoregional treatment response, number of tumors, tumor diameter and the amount of FFP given were not significantly different between the survived and deceased patients, PET-CT SUV_max_ value, the amount of erythrocyte solution (ES) as blood transfusion of red cells given and the tumor recurrence rate were significantly higher among the deceased patients (**Table 2**). In the univariate model, the effects of LDLT/DDLT, age, gender, FFP, ES, response to locoregional treatment, BMI, AFP, cold ischemia, tumor diameter, MVI and tumor number on survival time were not significant in Cox regression analysis while recurrence, ES, PET-CT SUV_max_ value and tumor differentiation had significant effects on survival.

**Table 2:** Factors Associated with Mortality in undergoing LT for HCC.

		Live				Died				p	
		Mean±sd/n-%			Median	Mean±sd/n-%			Median		
Age		62.4 ± 7.8			64.0	65.9 ± 7.6			67.0	***0.044***	^m^
Gender	Female	22		19.3%		3		13.0%		0.479	^X²^
	Male	92		80.7%		20		87.0%			
BMI		26.7 ± 3.7			26.0	26.3 ± 3.7			26.0	0.540	^m^
MELD		10.1 ± 6.6			8.5	9.7 ± 6.6			8.0	0.777	^m^
AFP (ng/ml)		46.7 ± 135.3			8.1	57.8 ± 70.1			23.7	0.125	^m^
Cold Ischemia (min)		145.8 ± 141.5			31.0	160.0 ± 175.0			30.0	0.897	^m^
Tm Diameter (mm)		27.3 ± 13.1			25.5	27.2 ± 11.0			30.0	0.988	^m^
Pet-CT		2.4 ± 2.0			2.0	3.5 ± 1.7			4.0	***0.004***	^m^
FFP (unit)	1	4		3.5%		1		4.3%		0.057	^X²^
	2	85		74.6%		12		52.2%			
	3	25		21.9%		10		43.5%			
ES (unit)	1	1		0.9%		0		0.0%		***0.012***	^X²^
	2	32		28.1%		4		17.4%			
	3	46		40.4%		5		21.7%			
	4	31		27.2%		7		30.4%			
	5	4		3.5%		7		30.4%			
Locoregional Treatment	no	80		70.2%		14		60.9%		0.380	^X²^
	yes	34		29.8%		9		39.1%			
MVI	no	73		64.0%		12		52.2%		0.285	^X²^
	yes	41		36.0%		11		47.8%			
Child-Pugh score	A	70		61.4%		13		56.5%		0.839	^X²^
	B	40		35.1%		9		39.1%			
	C	4		3.5%		1		4.3%			
Tumor Number (lesion)	1	77		67.5%		14		60.9%		0.707	^X²^
	2	17		14.9%		6		26.1%			
	3	19		16.7%		3		13.0%			
	4	1		0.9%		0		0.0%			
Tumor Differansiasion	Well	40		35.1%		5		21.7%		0.098	^X²^
	Moderately	59		51.8%		11		47.8%			
	Poorly	15		13.2%		7		30.4%			
Recurrence of tumor	no	108		94.7%		9		39.1%		***0.000***	^X²^
	yes	6		5.3%		14		60.9%			
LDLT		62		54.4%		13		56.5%		0.851	^X²^
DDLT		52		45.6%		10		43.5%			
Follow Up time (month)		55.2 ± 23.3			52.0	23.5 ± 15.4			16.0	***0.000***	^m^

BMI, Body mass ındex; AFP, Alpha Feto Protein; FFP, Fresh Frozen Plasma; ES, Eritrosit solution; MVI, Micro vascüler invasion; LDLT, Living donor liver transplantation; DDLT, Deceased donor liver transplantation.

^m^ Mann-whitney u test / ^X²^ Chi-square test.

We have shown statistically significant values in bold (p<0.05).

In the multivariate reduced model, cox regression analysis showed significant effects of recurrence, ES, locoregional treatment response and PET-CT on survival (**Table 3**).

**Table 3:** Uni and multivariable Cox proportional hazards model analysis for overall survival in undergoing LT for HCC.

Survival	Univariate Model			Multivariate Model		
	HR	% 95 CI	p	HR	% 95 CI	p
LDLT/DDLT	0.84	0.37 – 1.93	0.686			
Age	1.06	1.00 – 1.13	0.064			
Gender	1.38	0.41 – 4.66	0.600			
Meld	1.00	0.94 – 1.07	0.976			
Reccurens	13.39	5.76 – 31.14	***0.000***	12.65	5.32 – 30.08	***0.000***
FFP	2.16	0.98 – 4.74	0.055			
ES	2.35	1.45 – 3.79	***0.000***	2.35	1.46 – 3.78	***0.000***
Locoregional Treatment	1.32	0.57 – 3.07	0.512			
BMI	0.97	0.87 – 1.09	0.657			
AFP(ng/ml)	1.00	1.00 – 1.00	0.777			
Cold Ischemia	1.00	1.00 – 1.00	0.809			
TM Diameter	1.00	0.97 – 1.03	0.949			
MVI	1.65	0.73 – 3.73	0.233			
Child-pugh score	1.28	0.63 – 2.61	0.495			
Tumor Number	1.04	0.62 – 1.73	0.882			
Pet -CT(suv-max)	1.30	1.09 – 1.55	***0.004***	1.42	1.10 – 1.83	***0.007***
Tumor Differansiasion	1.93	1.05 – 3.53	***0.033***			
Cox Regression (Forward LR)						

BMI, Body mass ındex; AFP, Alpha Feto Protein; FFP, Fresh Frozen Plasma; ES, Eritrosit solution; MVI, Micro vascüler invasion; LDLT, Living donor liver transplantation; DDLT, Deceased donor liver transplantation.

We have shown statistically significant values in bold (p<0.05).

**Figure 1:**
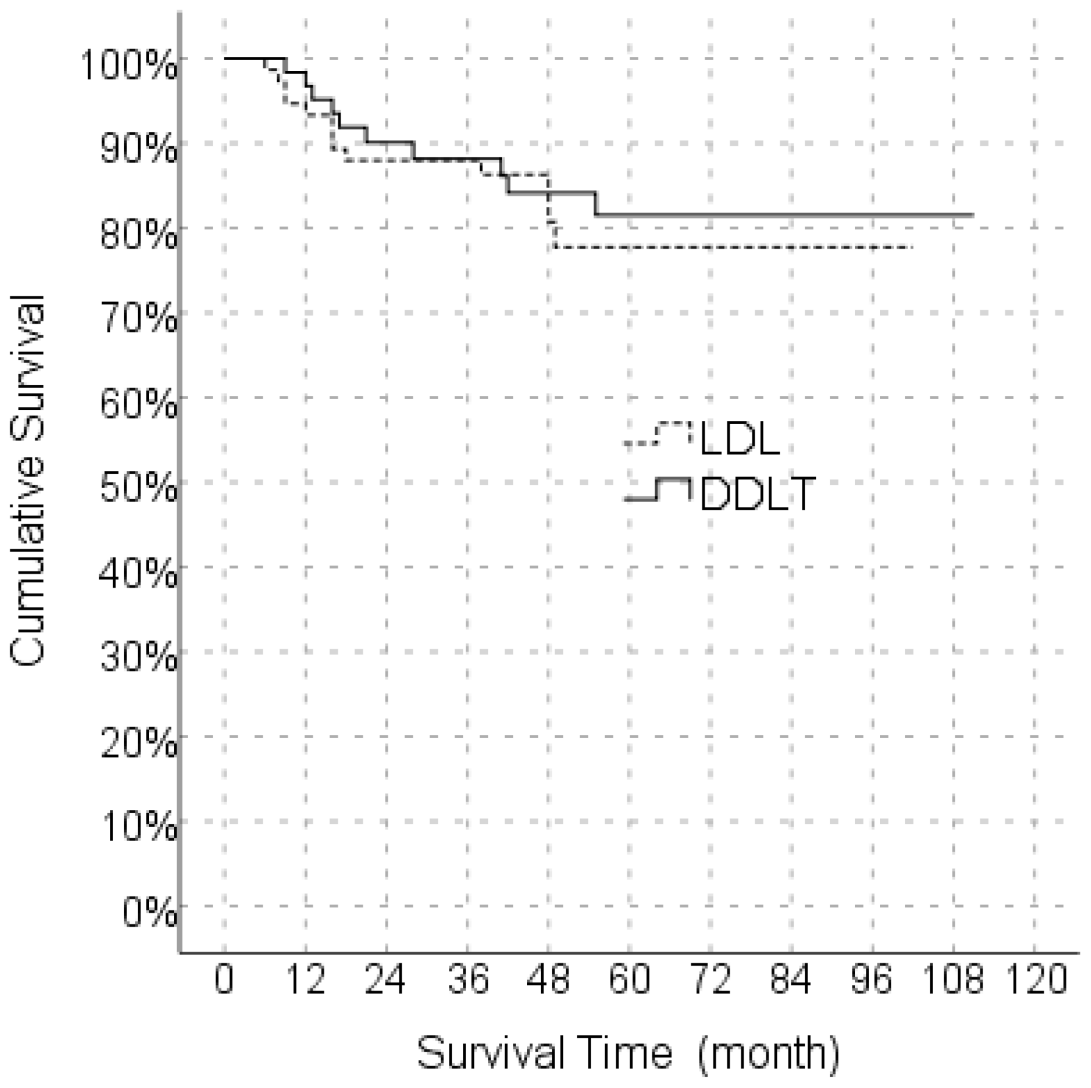
Comparison of Overall survival between LDLT and DDLT. LDLT, Living donor liver transplantation; DDLT, Deceased donor liver transplantation.

In the Kaplan Meier analysis, the predicted median survival time in the LDLT group was 85.6 months whereas the median predicted survival time in the DDLT group was 95.6 months, and the difference was not significant (**Figure 1**).

Age, sex distribution, BMI, tumor diameter and number, response to locoregional treatment, amount of FFP and ES given, and follow-up periods were not significantly different between the recurrent and non-recurrent groups. On the other hand, the MVI rate was significantly higher in the group with recurrence than in the group without the degree of tumor differentiation. The recurrence rate was higher in patients with low MELD scores and in patients with higher AFP value and cold ischemia time. Similarly, a linear correlation was observed between PET-CT SUV_max_ value and recurrence rate (**Table 4**).

**Table 4:** Multivariate analysis for recurrence in undergoing LT for HCC patients.

		Recurrence (no)				recurrence (yes)				p	
		Mean±sd/n-%			Median	Mean±sd/n-%			Median		
Age(years)		62.6 ± 7.8			64.0	64.8 ± 8.5			66.5	0.219	^m^
Gender	Female	21		17.9%		4		20.0%		0.826	^X²^
	Male	96		82.1%		16		80.0%			
BMI		26.6 ± 3.7			26.0	27.3 ± 3.7			27.0	0.405	^m^
Meld		10.4 ± 6.5			9.0	7.6 ± 6.5			5.5	***0.017***	^m^
AFP(ng/ml)		44.9 ± 133.8			6.8	69.5 ± 69.7			49.0	***0.000***	^m^
Cold Ischemia		136.5 ± 141.0			28.0	216.3 ± 166.2			210.0	***0.031***	^m^
Tm Diameter(max)		27.2 ± 13.0			25.0	27.5 ± 11.2			30.0	0.891	^m^
Pet-CT(suv-max)		2.4 ± 2.0			2.2	3.3 ± 1.7			4.1	***0.030***	^m^
FFP	I	4		3.4%		1		5.0%		0.828	^X²^
	II	83		70.9%		14		70.0%			
	III	30		25.6%		5		25.0%			
ES	I	0		0.0%		1		5.0%		0.865	^X²^
	II	31		26.5%		5		25.0%			
	III	46		39.3%		5		25.0%			
	IV	33		28.2%		5		25.0%			
	V	7		6.0%		4		20.0%			
Locoregional Treatment	(-)	83		70.9%		11		55.0%		0.156	^X²^
	(+)	34		29.1%		9		45.0%			
MVI	(-)	80		68.4%		5		25.0%		***0.000***	^X²^
	(+)	37		31.6%		15		75.0%			
Child-pugh score	I	70		59.8%		13		65.0%		0.849	^X²^
	II	43		36.8%		6		30.0%			
	III	4		3.4%		1		5.0%			
Tumor Number	I	81		69.2%		10		50.0%		0.153	^X²^
	II	19		16.2%		4		20.0%			
	III	16		13.7%		6		30.0%			
	IV	1		0.9%		0		0.0%			
Tumor Differansiasion	I	39		33.3%		6		30.0%		***0.039***	^X²^
	II	63		53.8%		7		35.0%			
	III	15		12.8%		7		35.0%			
LDLT		68		58.1%		7		35.0%		0.055	^X²^
DDLT		49		41.9%		13		65.0%			
Follow Up time		52.9 ± 24.4			50.0	32.2 ± 22.2			24.5	***0.001***	^m^

BMI, Body mass ındex; AFP, Alpha Feto Protein; FFP, Fresh Frozen Plasma; ES, Eritrosit solution; MVI, Micro vascüler invasion; LDLT, Living donor liver transplantation; DDLT, Deceased donor liver transplantation.

^m^ Mann-whitney u test / ^X²^ Chi-square test.

We have shown statistically significant values in bold (p<0.05).

**Figure 2:**
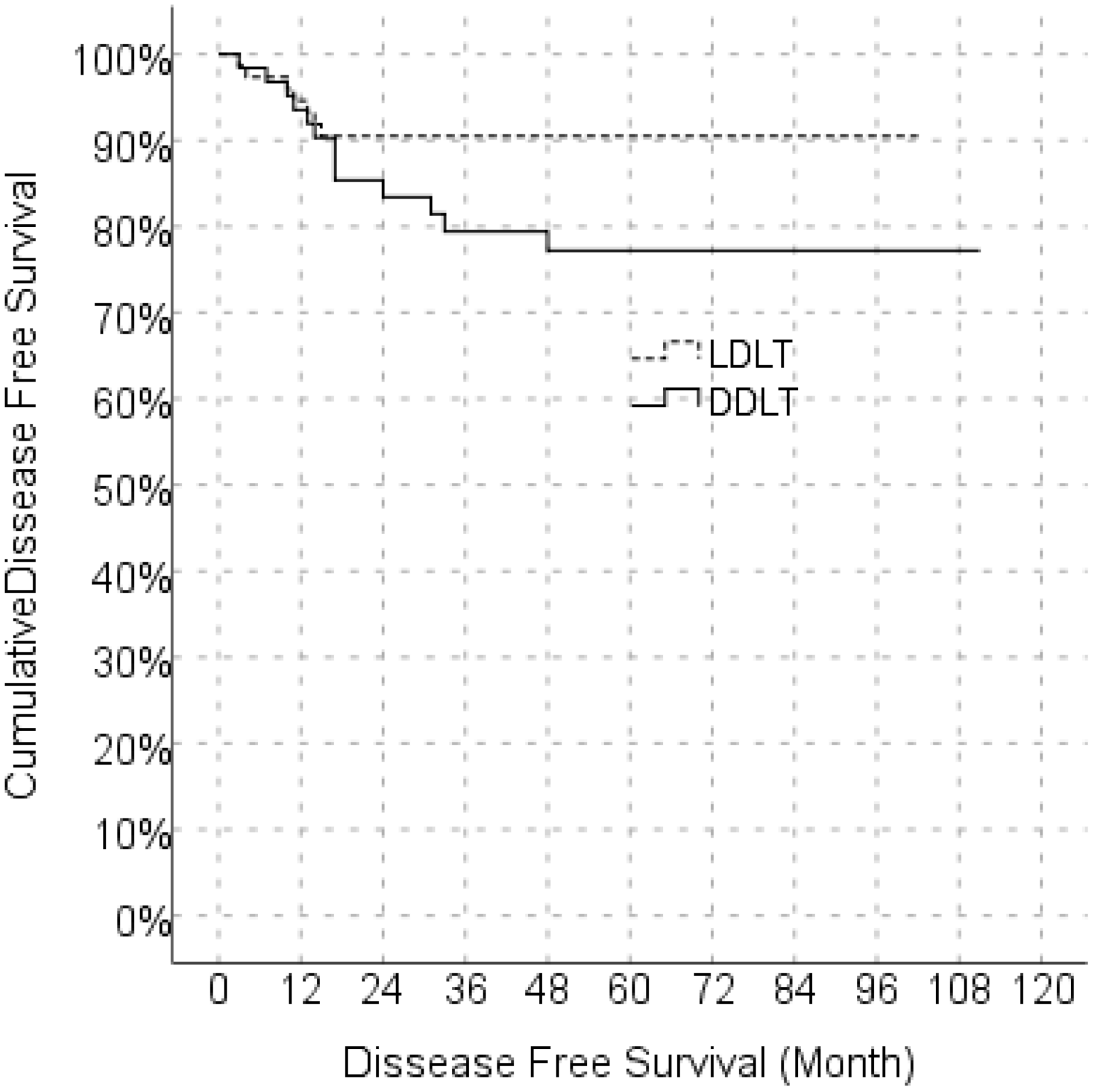
Comparison of disease free survival between LDLT and DDLT. LDLT, Living donor liver transplantation; DDLT, Deceased donor liver transplantation.

In the univariate model, LDLT/DDLT, age, gender, MELD score, response to locoregional treatment, FFP, ES, BMI, number of tumors and degree of differentiation did not have a significant effect on disease-free survival, while the effects of MVI, PET-CT SUV_max_ value and cold ischemia time on disease-free survival were significant. In the multivariate reduced model, only MVI had a significant effect on disease-free survival (**Table 5**). The predicted disease-free median survival time in the LDLT group (93.2 months) and the predicted median disease-free survival time in the DDLT group (90.3 months) were not significantly different. But 1,3 and 5 years cumulative OS for LDLT and DDLT were 93.3% and 96%, 88.0% and 88,1%, 77.6% and 81.5% respectively (**Figure 1**). But 1,3 and 5 years cumulative DFS for LDLT and DDLT were 94.6% and 93.5%, 90.5% and 79.4, 90.5% and 77.2% respectively (**Figure 2**).

**Table 5:** Uni- and multivariable Cox proportional hazards model analysis for recurrence-free survival in undergoing LT for HCC.

Dissease Free Survival	Univariate Model			Multivariate Model		
	HR	% 95 CI	p	HR	% 95 CI	P
LDLT/DDLT	2.220	0.885 – 5.568	0.089			
Age	1.036	0.974 – 1.103	0.257			
Gender	0.875	0.292 – 2.618	0.811			
Meld	0.926	0.846 – 1.014	0.098			
FFP	0.976	0.398 – 2.397	0.958			
ES	1.278	0.781 – 2.091	0.329			
Locoregional Treatment	1.894	0.784 – 4.575	0.156			
BMI	1.048	0.933 – 1.177	0.430			
AFP(ng/ml)	1.001	0.999 – 1.003	0.455			
Cold Ischemia	1.003	1.000 – 1.006	***0.035***			
TM Diameter	1.001	0.967 – 1.035	0.976			
MVI	5.643	2.049 – 15.540	***0.001***	5.643	2.049 – 15.540	***0.001***
Child-pugh score	0.928	0.416 – 2.072	0.856			
Tumor Number	1.521	0.945 – 2.446	0.084			
Pet -CT	1.207	1.001 – 1.457	***0.049***			
Tumor Differansiasion	1.706	0.892 – 3.266	0.107			
Cox Regression (Forward LR)						

BMI, Body mass ındex; AFP, Alpha Feto Protein; FFP, Fresh Frozen Plasma; ES, Eritrosit solution; MVI, Micro vascüler invasion; LDLT, Living donor liver transplantation; DDLT, Deceased donor liver transplantation.

We have shown statistically significant values in bold (p<0.05).

**Figure 3:**
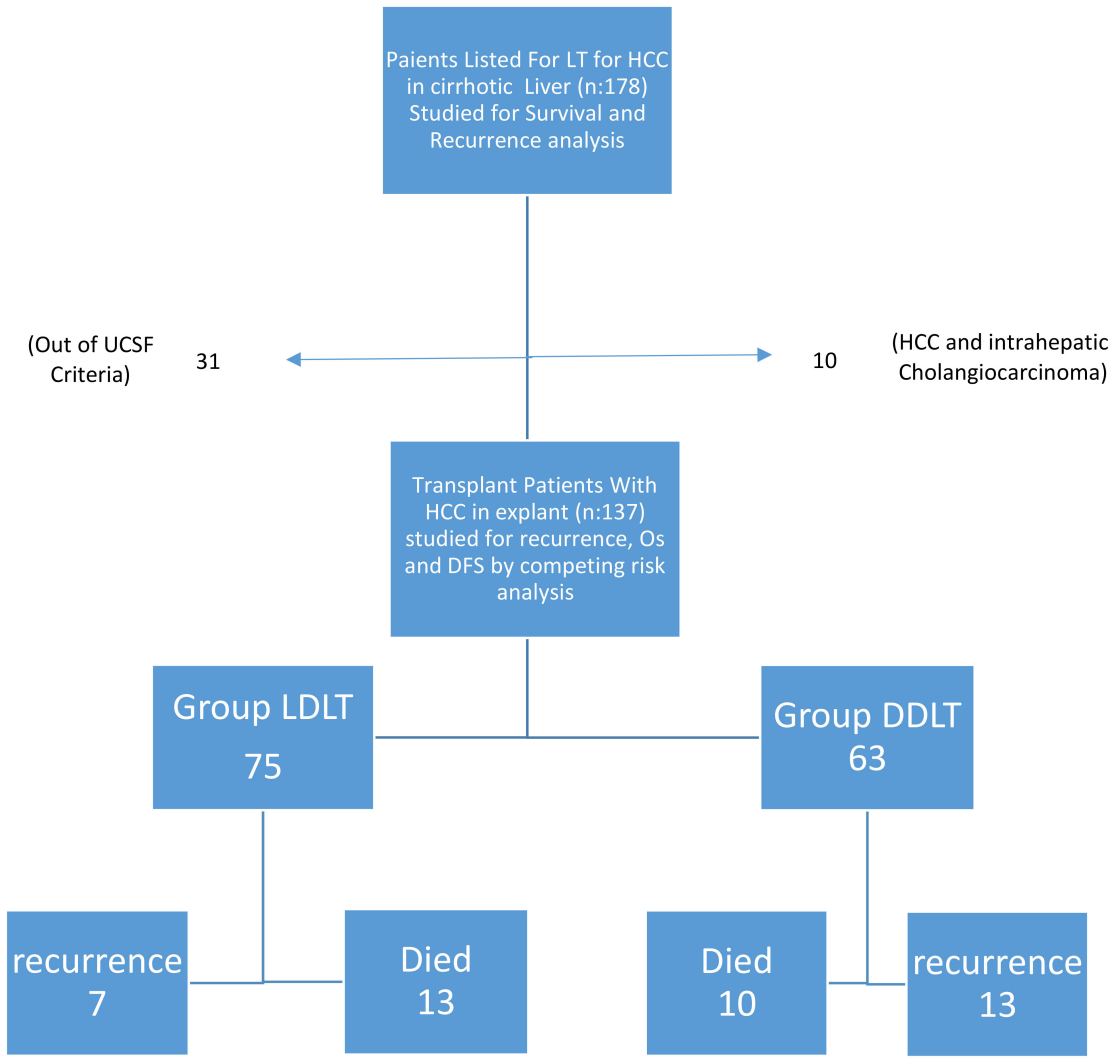
flow chart illustrating our. study populations.

When DDLT and LDLT were compared, there was no significant difference for gender, age, MELD score, AFP, PET-CT SUV_max_ value, tumor number and diameter, tumor differentiation degree, mortality rate, amount of FFP and ES given, Child score and MVI, while BMI rate, cold ischemia time, follow-up time and response rate to locoregional treatment were significantly higher in the DDLT group than in the LDLT group. Albeit not significant, the one-year recurrence rate in the LDLT was similar to that in the DDLT (5.4 vs. 6.5%, respectively), three- and five-year recurrence rates were higher in DDLT (20.6 and 22.8%, respectively) compared to LDLT (9.5 and 9.5%). We showed the study population as a flow chart in **Figure 3**.

## Discussion

The University of California Los Angles (UCLA) group reported it in a larger cohort of patients within UCSF but outside Milan, showing a 64% five-year survival rate (12). Upon the accumulation of LDLT cases performed in patients with HCC, the impact of DDLT over LDLT on the risk of recurrence of HCC has become a subject of considerable debate. The A2ALL cohort study reported that the differences between the two procedures for waiting time, pretransplant HCC management and tumor characteristics were responsible for the high recurrence in LDLT (5). İt should be kept in mind that HCC recurrence is the most common cause of death post-transplant. In some studies conducted in Asian centers, recurrence rates for HCC after LDLT were reported to range from 15 to 29% (13–15). It was also hypothesized that rapid regeneration might be associated with accelerated tumor cell growth. However, a few studies demonsrated such findings with even better recipient survival rates after LDLT, liver regeneration processes might be of limited relevance for HCC recurrence (16, 17). In fact, the overall controversial clinical results also point to confounders, other than on the recipient cancer risk. All of them may have impact on recurrence rates. Donor risk factors [i.e., prolonged warm or cold ischemia, graft recipient weight ratio (GRWR)], surgical parameters (duration of transplantation, medical management and transfusion policy) and the recipient risk (performans status, MELD score) are just a few adjunct variables (18).

LDLT using small grafts with GRWR of <0.8% was associated with lower overall and particularly tumor- free survival rates in patients with higher tumor burden outside Milan criteria. İn our study, the GRWR rate in all living donor grafts was between 0.8 to 1.0.

However in reality, when tumor characteristics were examined after recurrences in both modalities, no difference was found between DDLT and LDLT in terms of tumor biology after donor liver graft regeneration (14). Nevertheless, the main interest in that study was over whether the UCSF and Milan criteria used for DDLT should be used in LDLT. Since perioperative variables are normally included in intent to treat (ITT) analysis, it is obvious that the factors independent of operation type can also affect survival. ITT analysis only minimized pretransplant differences between LDLT and DDLT. Unlike ITT-OS, the studies dealing with post-LT OS excluded HCC patients with tumor-related death and tumor progression from the waiting list prior to DDLT, resulting in inevitable bias. One of the main concerns when LDLT is offered to patients with HCC is the fast tracking of transplantation and subsequent higher risk of posttransplant HCC recurrence. Previous studies showed that shorter waiting time may be associated with higher post-transplant HCC recurrence (19, 20). However, this was inconsistent with the positive effect of shorter waiting time on ITT OS explained above. Furthermore, due to tumor progression that exceeds criteria during the waiting time in DDLT regulation, the patients with biologically more aggressive HCC may be dropped from the waiting list, resulting in better survival and recurrence outcomes. The majority of studies comparing LDLT with DDLT proved that vascular invasion and poorly differentiated features are independent risk factors for an increased risk of recurrence after LT (21). MVI is independent risk factor for risk of recurrence after LT in our study. Despite our study showed similar survival outcomes, there were differences in 3 and 5 years DFS between LDLT and DDLT. High recurrence rates could be the reason for differences. Another reason might be inherent advantage with the shorter waiting times for candidates where a living donor is available. Goldaracena et al. demonstrated that patients who had a potential live donor at the time of listing had a higher survival rate (16). 3 and 5 years HCC recurrence rates were higher in DDLT compared to LDLT in our study. Consistent with other studies, the MVI rate, tumor differentiation grade, high AFP value, and long cold ischemia time were associated with high recurrence rates in our study. Furthermore, early recurrence within 2 years of undergoing surgery accounts for 70% of relapsed HCC patients, is almost incurable and has been related to terrible survival (22). Despite the implementation of strict criteria, HCC recurrence post LT remains up to 20%, occurring mostly within 2–3 years post LT (6). Our study reports HCC median recurrence rate post LT was 9.3% and 17.3% for LDLT and DDLT respectively. but early recurrence rate was no statiscically significant difference within two years both LDLT and DDLT. Apart from graft type, there are definite differences between LDLT and DDLT, such as shorter waiting time, quality graft with normal liver function, shorter ischemic time and the optimization of pre-transplant therapy, which may contribute to improved survival in patients undergoing LDLT. In our opinion, patients who have living donors should not wait for transplant other than the work up time for living donor. A randomized clinical trial would be best to resolve the controversy regarding the differences in outcomes between LDLT and DDLT for HCC patients, but this could prove difficult given the complex decision-making process and multidisciplinary approach involved in liver transplantation for HCC patients. However, there is no prospective study on this subject. There have been a systematic review and two meta-analyses on this subject so far. Liang et al. performed a meta-analysis with seven retrospective studies. They found the rate of disease-free survival and overall survival to be similar. The studies had poor general patient characteristics and low data quality scores. There was heterogeneity in critical considerations such as basic tumor characteristics and indication criteria, which did not provide strong evidence for the meta-analysis outcome (23). A large cohort, single-center study of more than 800 patients undergoing LT for HCC emphasized that in addition to tumor size, other clinicopathological parameters were useful and necessary to identify patients at lower risk of tumor recurrence (24). Graft availability in LDLT is unlimited and has no impact on waiting time. LDLT offers the opportunity for pre-transplant management and more optimized transplantation time to achieve considerably better possible outcomes. Basically, there is no significant difference between LDLT and DDLT for the post-transplant therapy management. It was reported that the surgical technique in LDLT does not comply with oncological surgery principles, and that strategies for preserving limited split liver volume such as natural inferior vena cava, longer bile duct, hepatic artery and portal vein in LDLT lead to the possibility of remnant tumor (25–27). However, in our study, the low recurrence and DFS rates in the LDLT group did not support this view. Due to low surgical mortality, optimal selection criteria and advances in reliable surgical methodology, LDLT has become a standard surgical procedure used for HCC. Furthermore, blood loss during liver transplantation is high due to intra-abdominal varices and portal hypertension as a result of end-stage liver failure in addition to the coagulation disorder associated with liver failure. Studies on the effects of allogeneic blood transfusion on tumor recurrence and survival after transplantation are rare, and disagreement remains on this subject (28). It was reported that blood loss and blood transfusion during hepatectomy for HCC is associated with worse overall and disease-free survival and higher recurrence rates. Perioperative blood transfusion was speculated to cause immunomodulation reactions. Indeed, it has a detrimental effect on survival and recurrence (29). However, immunosuppression is already added to post-LT patients. Therefore, the immunomodulatory effect may not be significant in patients with hepatectomy. Although the transfusion strategy in the present study did not include a strict study protocol, there was a significant relationship between the amount of erythrocyte suspension and death rate while the amount of FFP given and ES amount had no effect on survival in the univariate model. However, in the multivariate model, the amount of ES had significant effect on survival. Although FFP and ES were shown to be associated with oncological outcomes in other malignancies such as those of pancreas and colon, advanced studies are lacking for HCC. However, further subgroup analysis was not performed in patients receiving FFP due to the small cohort of our study. Similar to microvascular invasion/differentiation, glucose metabolism of HCC is associated with grade and aggressivity of the tumor. Therefore, the ^18^F-fluorodeoxyglucose positron emission tomography scan (FDG) of HCC can used a potential biomarker for the pre-transplant evaluation of the risk of recurrence after liver transplantation. In the present study, a significant relationship was observed between PET-CT SUV_max_ and death and reduced disease-free survival time, while a significant relationship was observed between total survival and PET-CT SUV_max_ in the univariate and multivariate model. The present study showed that although neither modality was significant predictor of overall and recurrence-free survival. Our study showed that although both modalities were not significant predictors of overall and recurrence-free survival, tumor differentiation, along with microvascular invasion, long cold ischemia time, higher ES amount given and elevated PET-CT SUV_max_ level were predictors of poor oncological outcomes. In fact, although the present study comparing HCC patients undergoing LDLT and DDLT did not clearly demonstrate OS benefit, it is important to assess the impact of these two modalities on DFS and recurrence. In regions with limited deceased donors, LDLT provides a better survival benefit for HCC patients who have more suitable tumor biology to reach DDLT. Although patient selection, waiting time, surgical management, liver graft suitability and structure and transplantation criteria were different in the two modalities, the survival rate was similar. Well-designed randomized controlled trials are needed to show whether LDLT or DDLT is more beneficial. In countries with no or limited access to a deceased donor organ pool for various reasons, LDLT has been developed with excellent outcomes for HCC recipients. Despite the idea that the high percentage of patients with tumors beyond the Milan criteria in LDLT may explain the high recurrence rates, the shortened waiting time may also reduce the rate of tumor recurrence and metastasis, helping to prevent the time-related risk of tumor progression. Post-transplantation prognosis in HCC patients especially in those beyond Milan criteria, should be based on measurable pre-liver transplantation situations and evaluated with external independent prospective cohorts.

Our study had limitations such as its retrospective nature, lack of strict study protocol for the transfusion strategy, and lack of cut-off values for biological markers such as AFP and PET-CT. However, the fact that it is a two-center study was important in the comparison of LDLT and DDLT within UCSF in terms of including the effect of PET-CT, cold and warm ischemia times and ES and FFP given. Sample size/power analysis was not performed for this study.

## Conclusion

Although the parameters reflecting tumor aggression such as vascular invasion and poorly differentiated features, high AFP level, increased PET-CT SUV_max_ value, and response to locoregional therapy, and prolonged cold ischemia time are risk factors for recurrence after liver transplantation, no significant difference was observed between LDLT and DDLT in terms of disease-free and overall survival rates. However, the recurrence rate was higher in DDLT than in LDLT, though not statistically significant. While there is less chance of cold ischemia time and better-quality grafts with minimal fatty changes, lower recurrence rates and similar survival rates can be achieved in LDLT compared to DDLT as long as biologically aggressive tumors are eliminated. We stated that owing to the significant differences between the Eastern and the Western worlds, a head-to-head comparison of LDLT vs DDLT activities performed in the same center instead of a comparison among international centers should be a better way for comparison accurately evaluating the survival benefit of live donation. Our study was designed to evaluate both an international activity in which centers polarized to an exclusive DDLT or LDLT activity were enrolled and a single-center activity in which both the experiences are commonly done.

## Data Availability

The raw data supporting the conclusions of this article will be made available by the authors, without undue reservation.
